# 4486 Assessing the Validity of an ICD-9 and ICD-10 Coding Algorithm for Identifying Cervical Premalignant Lesions Using Administrative Claims Data

**DOI:** 10.1017/cts.2020.167

**Published:** 2020-07-29

**Authors:** Jaimie Zhi Shing, Marie Griffin, James C Slaughter, Manideepthi Pemmaraju, Edward F Mitchel, Rachel S Chang, Pamela C Hull

**Affiliations:** 1 Vanderbilt University Medical Center; 2 Vanderbilt University School of Medicine

## Abstract

OBJECTIVES/GOALS: We compared the validity of an International Classification of Diseases, Clinical Modification (ICD) algorithm for identifying high-grade cervical intraepithelial neoplasia and adenocarcinoma in situ (together referred to as CIN2+) from ICD 9th revision (ICD-9) and 10th revision (ICD-10) codes. METHODS/STUDY POPULATION: Using Tennessee Medicaid data, we identified cervical diagnostic procedures in 2008-2017 among females aged 18-39 years in Davidson County, TN. Gold-standard cases were pathology-confirmed CIN2+ diagnoses validated by HPV-IMPACT, a population-based surveillance project in catchment areas of five US states. Procedures in the ICD transition year (2015) were excluded to account for implementation lag. We pre-grouped diagnosis and procedure codes by theme. We performed feature selection using least absolute shrinkage and selection operator (LASSO) logistic regression with 10-fold cross validation and validated models by ICD-9 era (2008-2014, N = 6594) and ICD-10 era (2016-2017, N = 1270). RESULTS/ANTICIPATED RESULTS: Of 7864 cervical diagnostic procedures, 880 (11%) were true CIN2+ cases. LASSO logistic regression selected the strongest features of case status: Having codes for a CIN2+ tissue diagnosis, non-specific CIN tissue diagnosis, high-grade squamous intraepithelial lesion, receiving a cervical treatment procedure, and receiving a cervical/vaginal biopsy. Features of non-case status were codes for a CIN1 tissue diagnosis, Pap test, and HPV DNA test. The ICD-9 vs ICD-10 algorithms predicted case status with 68% vs 63% sensitivity, 95% vs 94% specificity, 63% vs 64% positive predictive value, 96% vs 94% negative predictive value, 92% vs 89% accuracy, and C-indices of 0.95 vs 0.92, respectively. DISCUSSION/SIGNIFICANCE OF IMPACT: Overall, the algorithm’s validity for identifying CIN2+ case status was similar between coding versions. ICD-9 had slightly better discriminative ability. Results support a prior study concluding that ICD-10 implementation has not substantially improved the quality of administrative data from ICD-9.

